# Chemical and Mechanistic Prediction Analysis of Anti-obesity Properties of Guang Hawthorn (*Malus doumeri*) Leaves using Network Analysis

**DOI:** 10.2174/0113862073375434250529170324

**Published:** 2025-06-18

**Authors:** Lu Chen, Guibing Meng, Yu Pan, Lijun Yin, Yu Liu, Danna Huang, Li Jiang, Mingsheng Lan, Chao Luo, Wuwei Wu

**Affiliations:** 1 Guangxi Medicinal Botanical Garden, Nanning 530023, P.R. China;; 2 National Engineering Research Center for Southwest Endangered Medicinal Materials Resources Development, Nanning, 530023, P.R. China;; 3 Department of Pharmacy, Shanghai Mental Health Center, Shanghai Jiaotong University, School of Medicine, Shanghai, 200030, P.R. China

**Keywords:** Hawthorn (*Malus doumeri*) leaf, phlorizin, sieboldin, kumatakenin β-7-O-glucoside, anti-obesity, network pharmacology

## Abstract

**Introduction:**

Obesity is a global health issue linked to metabolic disorders and cardiovascular diseases. Guang hawthorn (*Malus doumeri*) leaves have been traditionally used for medicinal purposes, but their bioactive compounds and anti-obesity potential remain underexplored.

**Methods:**

This study extracted compounds from *M. doumeri* leaves using 70% ethanol and ethyl acetate. The extracts were administered to high-fat diet-induced obese rats. Serum levels of total cholesterol (TC), triglycerides (TG), high-density lipoprotein cholesterol (HDL-C), and low-density lipoprotein cholesterol (LDL-C) were measured. The chemical composition of the extracts (HML) was analyzed using chromatography, NMR, and mass spectrometry. Network pharmacology and enrichment analyses were conducted using R and Cytoscape to identify compound-target interactions.

**Results:**

Rats treated with high-dose extracts showed significantly reduced TC, TG, and LDL-C levels and increased HDL-C (all *p* < 0.05). Three major compounds-phlorizin, sieboldin, and kumatakenin β-7-O-glucoside-were identified. A total of 272 overlapping targets and 32 core targets were found between compound-related and obesity-related gene sets. Functional analysis linked these targets to phosphorylation, apoptosis, cell proliferation, and kinase regulation.

**Discussion:**

The anti-obesity effects of *M. doumeri* may be mediated by modulation of the PI3K-Akt and FoxO signaling pathways, as well as proteoglycan biosynthesis. These pathways are associated with metabolic regulation and obesity-related changes.

**Conclusion:**

*M. doumeri* leaf extracts demonstrate anti-obesity potential through multi-target and multi-pathway mechanisms, particularly *via* sieboldin and kumatakenin β-7-O-glucoside. These findings support their potential as natural therapeutic agents for obesity management.

## 1 INTRODUCTION

Obesity is an escalating worldwide public health problem [1]. According to the World Health Organization estimates, in 2022, 2.5 billion adults (18 years and older) were overweight. Of these, 890 million were living with obesity [2]. Over 1.9 billion adults were reported to be overweight (clas sified as a body mass index (BMI) ≥ 25 kg/m2), and 650 million were in the obese range (with BMI ≥ 30 kg/m^2^) in 2016 [1]. With the rising prevalence of obesity, there is a gradual increase in the mortality rates and risk of chronic diseases. These include cardiovascular disease, cancers, hypertension, stroke, neurodegenerative diseases, type II diabetes, fatty liver disease, and asthma [1, 3]. Jaacks *et al.* [4] proposed a four-stage model for tackling this major problem. They proposed that 30 large countries with approximately 77.5% of the world's population should be the research subjects and indicated that most countries are in the first three stages, and it is only during the fourth stage that the prevalence of obesity may decline.

Obesity is primarily a disorder of lipid metabolism [5], which is characterized by an increase in the size and/or number of fat cells in an individual’s adipose tissue [6]. Recent research indicates that obesity is closely related to the activation of the AMPK/PGC1 pathways [7], increased brown adipocyte tissue (BAT) deposition [8] as well as activation of the PI3K-Akt-mTORC1 pathways and negative regulation of autophagy within adipose tissues [9, 10].

In terms of drugs to combat obesity, in the past, dexfenfluramine (Redux), sibutramine (Meridia, Reductil), orlistat (Xenical) and rimonabant (Acomplia) have been used, but of these only orlistat is available in the UK obesity management and has not been withdrawn from the market [11]. Currently, the Food and Drug Administration (FDA) has approved orlistat, locaserin, phentermine/topiramate (extended release) and naltrexone/bupropion (extended release) or a fixed combination of bupropion/naltrexone and liraglutide for long-term use [11, 12]. Although anti-obesity agents can be used to treat obesity and possess huge potential markets, their side-effects limit their use. Consequently, we are focused on finding potentially safe, effective, cost effectiveness, and easily available herbal medicines with maximal anti-obesity potential but with minimal side-effects to fill this void [13, 14].

The big hawthorn leaf of Guangxi originates from *Malus doumeri* (Bois) *Chev*, which belongs to the genus *Malus* of the *Rosaceae* family [15]. It is commonly used in Taiwan and southern China instead of medicinal hawthorn, which originated from *Crataegus pinnatifida* Bge. and Var major N. E. Br [15, 16]. Hawthorn (*Crataegus*) leaves, a staple in traditional Chinese medicine, have been widely employed to address a range of health conditions. Specifically, they are recognized for their potential in ameliorating hyperlipidemia, managing heart failure symptoms, promoting blood circulation to reduce stasis, alleviating chest discomfort, and improving cognitive function by mitigating forgetfulness and dizziness. [17]. In order to deeply explore the anti-obesity mechanism of *Crataegus pinnatifida* leaves and effectively extract their active components, modern extraction techniques have become particularly important. In recent years, various efficient extraction methods have been applied to the extraction of active ingredients from traditional Chinese medicinal materials, including but not limited to sub/supercrit-ical water extraction [18], which significantly improves extraction efficiency and component purity by adjusting the temperature and pressure conditions of water; microwave-assisted extraction [19], which utilizes the rapid heating characteristics of microwaves to accelerate the release of intracellular substances; molecular distillation technology [20], which achieves effective separation of high-boiling-point and thermosensitive components through precise control of temperature and pressure; and infusion extraction [21], which improves the yield and purity of target components by optimizing the solvent system and extraction conditions. The application of these methods not only enriches the extraction techniques for active ingredients in traditional Chinese medicinal materials but also provides technical support for in-depth research on the anti-obesity properties of *Crataegus pinnatifida* leaves. Flavonoids are the major bioactive compounds of hawthorn leaves, and they possess anti-inflammatory, anti-oxidant, and lipidemic-lowering properties [22]. Compared to the leaves of *Crataegus*, those of *M. doumeri* have been studied rarely and are consumed frequently as a healthy and beneficial beverage [23]. At present, phlorizin is thought to be the major active component of *M. doumeri* leaves with proven anti-inflammatory, anti-oxidant, anti-diabetic, and anti-tumor activities [24, 25]. The hypo-lipidemic activities *M. doumeri* has not been fully explored, and this study was undertaken to resolve this. Network pharmacology, coupled with systematic and holistic characteristics, has been widely used as a promising method to explain the complex interactions between herbal medicines and diseases, and these have proven to be beneficial to us in our understanding of the usefulness of these natural products. The research conducted by Saeid Maghsoudi *et al.* has demonstrated that drug repositioning and chemical genomics can facilitate the identification of potential candidate drugs for therapeutic targets, thereby predicting drug toxicity [26]. Additionally, Fang *et al.* have emphasized the effectiveness of a new strategy based on network pharmacology for exploring the mechanism of action of Chinese herbal medicines from a holistic perspective [27]. Their use may help us in the future to find potential new effective herbs [28, 29]. The whole study process is shown in Fig. (**1**).

## 2 MATERIALS AND METHODS

### 2.1 Chemicals and Reagents

All solvents were analytical grade (China National Pharmaceutical Group Chemical Reagent). Biochemical kits for total cholesterol, triglyceride, LDL-c, and HDL-c were procured from Nanjing Jiancheng Bioengineering Research Institute. Tween 80 and propylene glycol were from Tianjin Fuyu Fine Chemical I Co., Ltd. Cholesterol and sodium cholate were from Solaibao Biotechnology Co., Ltd. Atorvastatin calcium tablets were from Zhejiang Xindonggang Pharmaceutical Co., Ltd., (batch number: 20140813, 10mg/tablet).

### 2.2 Equipment/Instruments

A rotary evaporator (RE-52, Shanghai Yarong Biochemical Instrument Factory), high performance liquid chromatograph system (Breeze, Waters), ultraviolet detector (TU-II, Shanghai Kezhe), magnetic mass spectrometer (MMS; MAT-95, Finigan), nuclear magnetic resonance (NMR) instrument (ANCE II-600, Bruker company), micro-quadrupole time-of-flight mass spectrometer (Q-TOF; Micromass, UK), electronic balance (CP114, Ohaus), melting point measuring instrument (XT-4A, Shanghai Jinhe), SCILOGEX desktop centrifuge (D3024, Cello Czech, USA) and microplate reader (DNM-9606, Shanghai Kehuai) were the instruments and their manufacturers used in this study.

### 2.3 Extraction of Plant Materials

The mature leaves of *M. doumeri* (Bois) Chev. were purchased from Napo, Guangxi, and identified by Prof. Xindong Nong from Guangxi Botanical Garden of Medicinal Plant. A representative specimen (voucher number: 4510261311 23034LY) has been deposited in the Guangxi herbarium of the Traditional Chinese Medicine Institute for further reference. The extraction of leaf components was conducted as follows: A total of 5 kg of *M. doumeri* leaves were subjected to two sequential extractions using 70% ethanol in a reflux system, each lasting 2 h. HML was recovered by using a rotary evaporator at 60°C, and the final extract, which weighed approximately 1.5 kg, was subsequently stored at 4°C.

### 2.4 Extraction, Separation and Analysis

30 L of purified water was added to 1.5 kg HML and heated to dissolve, and then the mixture was refluxed. This was followed by the same amounts of petroleum ether, chloroform, ethyl acetate, and n-butanol, and each solvent were extracted 3 times, and then concentrated to obtain the polarity of each extract fraction. The chemical composition of the extracts was analyzed using the instruments described above..

### 2.5 Treatment of Animals

Male Sprague Dawley rats, aged 8 weeks and weighing 200±20 g, were purchased from the Medical Laboratory Animal Center of Guangxi Medical University (qualification No. SCK GUI 2014-0002). Fifty rats were fed under a controlled room temperature (22±2°C) and air relative humidity (65%±5%), and the animals were allowed free access to food and drinking water. The basic chow was also purchased from the Medical Laboratory Animal Center of Guangxi Medical University. This study was approved by the Animal Ethics Committee of China Pharmaceutical University (No. CPU-PCPK-S1010354). The rats were acclimatized for 7 days before the experiment and divided randomly into 5 groups (*n*=10/group) as follows: (1) normal diet (ND), (2) high fat diet (HFD), (3) high fat diet and 8.33 mg/kg of atorvastatin (HFD + ATV, i.g.), (4) high fat diet and 5 mg/kg of HML (HFD + HML-L, i.g.), and (5) high fat diet and 10 mg/kg of HML (HFD + HML-H, i.g.), lasting for 4 weeks. The HFD used consisted of 20% sucrose, 15% lard (*w/w*), 1.2% cholesterol, 0.2% sodium cholate, 10% fresh egg solution, and 53.6% normal chow [30]. The sample size of the animal and the dosage were determined by our previous work [31]. All high-fat feeds used the equal incremental method to mix sodium cholate, cholesterol, sucrose, and basic chow in a stepwise manner. Lard was melted at high temperatures and mixed with egg liquid, and then mixed with the feed by using a spray system. Finally, the mixture was passed through a granulator to make rod-shaped pellets, which were then blow-dried at 80°C for 8 hours. The five groups of rats were fed for 30 days using the above protocols. The body weights of all animals were measured after 0, 1, 2, 3 and 4 weeks.

### 2.6 Serum Sample Collection

After the last gavage, followed by fasting and but allowed access to water for 16 h, the rats were anesthetized by intraperitoneal injection of 0.5% pentobarbital at a dose of 0.1 mL/100g. After the rats entered a state of deep anesthesia, a 5 mL syringe was used for blood collection by cardiac puncture. The blood samples were maintained in a water bath at 37°C for 30 min, subsequently centrifuged at 3500 rpm/min for 15 min to separate the serum. The serum was then aliquoted to prevent repeated cycles of freezing and thawing, thereby ensuring sample integrity. Samples were stored at -20°C.

### 2.7 Biochemical Indicator Analysis

The serum levels of triglycerides (TG), total cholesterol (TC), low-density lipoprotein cholesterol (LDL-C), and high-density lipoprotein cholesterol (HDL-C) were measured from kits purchased from Nanjing Jiancheng Bioengineering Institute and following the manufacturers’ protocols.

### 2.8 Collection of Data Relating to Anti-obesity Properties of HML

All predicted targets of HML were obtained from the PharmMapper database. The GeneCards database was also employed in collecting known pathological targets for obesity. Subsequently, the targets from HML were matched to pathologic targets of obesity, and then the therapeutic targets of human neutrophil lipocalin (HNL) against obesity were selected.

### 2.9 Construction of PPI Networks, Hub Targets, and Cluster Analysis

We further analyzed the target-target interactions on the intersection targets by using the STRING database. Protein interactions with a minimum required interaction score of more than 0.9 and that are able to hide disconnected nodes in the network were selected in the designed settings after eliminating duplicates. The results were imported into Cytoscape (v3.8.0) to construct protein-protein interaction networks. The network analyzer in Cytoscape was utilized for analyzing topological parameters of the mean and maximum degrees of freedom in the PPI network of *M. doumeri* leaf extract compounds with anti-obesity properties. The core targets were screened according to a previously reported method [32]. The top 10 core targets were subsequently determined by the cytoHubba algorithm, with the MOCDE plugin being used for cluster analysis.

### 2.10 GO and KEGG Pathway Enrichment Analyses of Core Targets

The DAVID database (https://david.ncifcrf.gov/) was utilized for analyzing the GO biological functions and KEGG pathways. The core targets were imported into the DAVID database as well as the official gene symbols and gene lists. The results were analyzed using GraphPad Prism 9.0 software.

### 2.11 Statistical Analysis

The quantitative data are presented as means ± standard deviations (SD). The experimental data were analyzed by one-way analysis of variance (ANOVA) with the Duncan’s *post hoc* test using GraphPad Prism 9.0. The *R* software, version 3.6.2, was used for drawing the graphs. The results of the animal study were assessed by a third-party assessor who was blinded to the groups.

## 3 RESULTS

### 3.1 Identification of Active Ingredients in Ethyl Acetate

Nebeg *et al.* [33] reported that several flavonoids were present in the ethyl acetate extracts of *Thapsia garganica* leaves, and these have anti-obesity properties. This prompted us to conduct an in-depth analysis of the ethyl acetate extracts of *M. douumeri* leaves. Firstly, the ethyl acetate extract was dissolved in methanol, and the eluent capacity and its gradient ratio were determined by thin layer chromatography. Then, the extract was subjected to silica gel column chromatography, a macro-porous resin column, a Sephadex LH-20 gel, and a combination of several other multiple chromatographic techniques. Lastly, the isolated components were analyzed and identified by NMR and mass spectrometry.

Firstly, we obtained a light-yellow powder from the ethyl acetate extracts of *M. doumeri* leaves. The first compound was identified by MS, NMR, and a comparison with those in the literature. MS gave an m/z for compound A of 435.1296 [M-H]- and the chemical formula of C_21_H_24_O_10_, and an unsaturation value of 10. After using deuterated methanol to dissolve the powder, the NMR carbon and hydrogen spectral data obtained were as follows: ^13^C-NMR (151 MHz, CD_3_OD) δC : 132.4 (C-1), 129.0 (C-2,6), 114.6 (C-3,5), 154.9 (C-4), 105.3 (C-1′), 160.9 (C-2′), 94.0 (C-3′), 164.4 (C-4′), 96.9 (C-5′), 166.1 (C-6′), 205.1 (C-O), 45.6 (C- α), 29.4 (C -β), 100.6 (C-Glu-1), 73.3 (C-Glu-2), 76.9 (C-Glu-3), 69.6 (C-Glu-4), 77.1 (C-Glu-5), 61 (C-Glu-6). ^1^H-NMR (500 MHz, CD_3_OD) δH : 3.38 (2H, m, 2H-α), 2.86 (2H, t, J=7.1, 2 H- β), 7.05 (2H, d, J=8.5 Hz, H-2, H-6), 6.67 (2H, d, J= 8.5 Hz, H-3, H-5), 6.17 (1H, d, J= 2.3 Hz, H-3′), 5.95 (1H, d, J=2.2Hz, H-5′), 5.03 (1H, d, J= 7.3 Hz, H-Glu-1), 3.41-4.89 (4H, overlapped, H-Glu-2, 3, 4, 5), 3.89 (1H, dd, J = 2.0, 12.0 Hz, H-Glu-6α) and 3.70 (1H, dd, J= 5.4, 12.1 Hz, H-Glu-6β). According to the ^13^C-NMR measurements, there were 19 carbon signal peaks, and the heights and peak areas of two of these were 114.6 and 129.0, which were double the values obtained for the other signals, indicating that the signals contained two carbons, respectively.

From the chemical shift value of 205.1, we speculated that a carbonyl group was present. Based on the signals of 61.0, 69.6, 73.3, 76.9, 77.1, and 100.6 obtained, we implied that there was a six-carbon sugar structure present and that the signals 29.4 and 45.6 may be those of alkyl carbon. In addition, based on the degree of unsaturation combined with the number of carbon atoms, the compound may have two benzene rings. According to the ^1^H-NMR measurements, we postulated that there were 1, 2, 4 and 6 tetra-substituted benzene rings by comparing with the signals of δH: 6.17 (1H, d, J= 2.3 Hz), 5.95 (1H, d, J=2.2 Hz) and δH: 7.05 (2H, d, J=8.5 Hz), 6.67 (2H, d, J= 8.5 Hz). W, and further speculated that there was a para-substituted benzene ring. It was also speculated that the δH: 5.03 (1H, d, J = 7.3 Hz) signal obtained was from the end matrix proton signal of the sugar, and the coupling constant (J=7.3 Hz) indicated that the sugar was likely to be in the beta configuration. Finally, the 1H-NMR and ^13^C-NMR data of compound A were consistent with it being dihydrochalcone-2′-β-D-glucopyranoside (phlorizin) by a literature comparison, and so it was identified as such [34].

The light-yellow powder was further dissolved in deuterated methanol to identify a close companion to compound A. Using phloridzin as the basis of the analytical process, a hydroxyl group was seen at position 3 and the data from MS, ^13^C-NMR and ^1^H-NMR changed as follows: ^13^C-NMR (151 MHz, pyridine) δC : 133.8 (C-1), 117.2 (C-2), 147.1 (C-3), 145.3 (C-4), 116.6(C-5), 120.0 (C-6), 106.2 (C-1′), 162.4 (C-2′), 95.5 (C-3′), 166.5 (C-4′), 98.3 (C-5′), 167.7 (C-6′), 205.7 (C-O), 46.1 (C -α), 30.4 (C -β), 102.5 (C-Glu-1), 74.7 (C-Glu-2), 79.0 (C-Glu-3), 70.9 (C-Glu-4), 79.2 (C-Glu-5), 62.1 (C-Glu-6). And ^1^H-NMR (500 MHz, pyridine) δH : 3.49 (2H, m, 2H-α), 3.32 (2H, t, J=7.1, 2 H -β), 7.21 (1H, dd, J=8.0, 1.9 Hz, H-6), 7.40 (1H, d, J=8.0 Hz, H-5), 7.17 (1H, d, J= 2.1 Hz, H-2), 6.85 (1H, d, J= 2.2 Hz, H-3′), 6.00 (1H, d, J=2.2Hz, H-5′), 4.59 (1H, d, J= 8.9Hz,H-Glu-1), 4.61-4.66 (2H, m, H-Glu-2, 3,), 4.66-4.72 (2H, m, H-Glu-4, 5), 4.26 (1H, dd, J = 5.2, 12.0 Hz, H-Glu-6α) and 4.20 (1H, dd, J= 7.0, 12.1 Hz, H-Glu-6β). When the data were compared to those reported by Guernevé *et al.* [35] for 3-hydroxy-phloridzin (sieboldin), the results were consistent and thus compound B was identified as such.

Finally, after recrystallization, yellow needle crystals were obtained after the raphides were dissolved in deuterated methanol. MS gave an m/z for compound C of 415.1 [M-H]- and the chemical formula of C_21_H_20_O_9_, and an unsaturation value of 12. The MS, 13C-NMR and 1H-NMR data changed as follow: ^1^H-NMR (600 MHz, pyridine-d5): 6.06 (1H, s, H-3), 6.56 (^1^H, d, J= 2.2 Hz, H-6), 6.60 (1H, d, J= 2.4 Hz, H-8), 7.01 (^3^H, m, H-2', 6',5), 6.16 (2H, d, J= 9.0 Hz, H-3', 5'), 4.55 (1H, d, J= 7.7 Hz, H-1''), 3.57 (1H, m, H-6''a), 3.53 (1H, m, H-6''b), 3.43-3.52 (4H, sugar protons). ^13^C-NMR (150 MHz, pyridine-d5): 164.4 (C-2), 104.8 (C-3), 178.0 (C-4), 129.1 (C-5), 108.7 (C-6), 161.2 (C-7), 105.1 (C-8), 159.4 (C-9), 126.3 (C-10), 129.1 (C-1'), 131.6 (C-2'), 109.3 (C-3'), 160.0 (C-4'), 126.3 (C-5'), 131.4 (C-6'), 99.2 (C-1''), 75.1 (C-2''), 77.3 (C-3''), 70.8 (C-4''), 79.1 (C-5'') and 62.2 (C-6''). When the data were compared to those reported by Feng *et al.* [36] for kumatakenin β-7-O-glucoside, the results were consistent, and thus, compound C was identified as such. The diagrammatic representation of the structures of the three compounds are shown in Fig. (**2**).

### 3.2 Weight Trends of Rats in the Five Groups Over a Period of 4 Weeks

Based on the findings from the experimental animals, it was observed that the consumption of HFD could lead to an increase in body weight. As illustrated in Table **1**, there were no significant differences in the initial body weights of mice across the five dietary groups prior to the commencement of the 4-week dietary intervention. Following the 4-week period, mice in the HFD + ATV, HFD + HML-L, and HFD + HML-H groups exhibited a reduction in their final body weights compared to those in the HFD group, with statistical significance at *p* < 0.001. No rats were excluded from this study.

### 3.3 Serum Lipid Levels

There is a direct correlation between obesity and alterations in blood lipid levels. Consequently, an investigation was conducted to evaluate the impact of HML on lipid metabolism in mice with obesity induced by the HFD group. This involved measuring the levels of TG, TC, LDL-C, and HDL-C. In the model group, the activities of TG, TC, and LDL-C were significantly increased in serum (*p* < 0.01). HFD + ATV, HFD + HML-H treatment statistically decreased DSS-induced activation of TG, TC, LDL-C, and HDL-C in serum (*p* < 0.01, *p* < 0.05, respectively) (Fig. **3**). However, TG, TC, and LDL-C levels in serum showed a decreasing trend after HFD + HML-L treatment. Additionally, HDL-C levels in serum showed an increasing trend after HFD + HML-L treatment. However, these changes were not statistically significant.

### 3.4 The Results of Predictive Analysis

A total of 641 targets were found for the 3 bioactive compounds identified in the ethyl acetate HML by using the Uniprot and PharmMapper databases. In addition, 8756 pathological targets were gathered from the GeneCards database. *R* software was then employed to analyze the therapeutic targets of the anti-obesity properties of HML and 272 were found.

### 3.5 The PPI and Compound-Targets-Disease Networks

The 272 therapeutic targets were imported into the STRING database, and the results with confidence scores >0.9 were selected while hiding the unconnected targets. The results were then introduced into Cytoscape (v3.8.0) to construct protein-protein interaction networks. Finally, 42 unrelated therapeutic targets were removed, and 230 connected nodes and 785 edges were saved in the loop network (Fig. **4**).

### 3.6 Clustering and Core Target Analysis in PPI Networks

Cluster and core target analysis were used as the basis for PPI networks by using the MCODE and the cytoHubba algorithms in Cytoscape software. 11 clusters were obtained, and the first red cluster showed the largest score (11.00) with 19 nodes and 99 edges. The top 10 ranked of the 8 algorithms of cytoHubba are shown in Table **2**, and the degree algorithms of cytoHubbawere found to work the best. The calculated maximum degree value was 41, and the doubled average degree value was 13.65 based on the degree algorithms. Therefore, the range of core targets was from 13.65 to 41 when the 32 core targets were screened (Fig. **5**).

### 3.7 GO and KEGG Pathway Enrichment Analysis of Core Targets

32 Hub genes were imported into the DAVID database in order to analyze the GO functions and KEGG pathways. 14 biological processes, 7 molecular functions, and 14 screened cell components were enriched with P-values of less than 0.05. The biological processes involved included peptidylserine phosphorylation, peptidyl-tyrosine autophosphorylation, negative regulation of apoptotic processes, regulation of cell proliferation, innate immune responses, positive regulation of the ERK1 and ERK2 cascades, cell migration, protein serine/threonine kinase activities, Ras protein signal transduction, peptidyl-threonine phosphorylation, epidermal growth factor receptor signaling pathway, positive regulation of cyclase activity, transmembrane receptor protein tyrosine kinase signaling pathway, positive regulation of protein kinase B signaling and growth hormone receptor signaling pathway.

Overall, the processes were related to various phosphorylation events, cell proliferation, apoptosis and various kinase activities, and other process. These processes are connected to the extrinsic components of cytoplasmic side of plasma membrane, cytosol, nucleus, cell cortex, mid-body, fibrinogen complex, cytoskeleton, lamellipodium, focal adhesion, platelet alpha granule, endosome, spindle midzone, cyclin-dependent protein kinase holoenzyme complex, chromosome and telomeric region. In addition, molecular function analysis showed that they are also related to ATP binding, non-membrane spanning protein tyrosine kinase activity, receptor binding, nitric-oxide synthase regulator activity, protein serine/threonine kinase activity, receptor signaling protein tyrosine kinase activity and structural molecule activity.

Our subsequent analysis revealed that the molecular signaling pathways of 32 core targets, which are implicated in the anti-obesity effects of HML, are intricately linked to proteoglycans in cancer, progesterone-mediated oocyte maturation, pathways in cancer, viral carcinogenesis, cell cycle, hepatitis B as well as the FoxO, prolactin and PI3K-Akt signaling pathways (Fig. **6**).

## 4 DISCUSSION

Hawthorn leaves are a well-known form of Chinese traditional medicine and belong to the genus *Crataegus* of the rose family [17]. Hawthorn leaves, flowers and fruits have demonstrated efficacy in treating hyperlipidemia and the flavonoids were speculated to act as active ingredients [37]. Earlier phytochemical studies had isolated approximately 30 flavonoids from leaf extracts of *Crataegus* [17]. However, the big hawthorn from Guangxi in China which is *M. doumeri* (Bois) Chev and belongs to genus *Malus* of *Rosaceae* family is the subject of this study [15]. To our knowledge, there was a research gap on the chemical components of this type of plant and its ability to combat obesity.

In this study, the effects of HML on blood lipid metabolism will be confirmed by serum biochemical indicators. Hyperlipidaemia is one of the abnormal lipid disorders of lipid metabolism dysfunction, the increased LDL-C or decreased HDL-C content is closely related to heart and vascular diseases [38], and serum TC and TG contents are crucial for the improvement of hyperlipidaemia [39-40]. Dyslipidaemia could aggravate the progression of corpulence syndrome by stimulating inflammatory cytokines, inducing IR, and impairing glucose metabolism, and dyslipidaemia is another prominent feature of obesity [41]. In comparison, atorvastatin is primarily used for the treatment of dyslipidemia and the prevention of cardiovascular disease. The present results suggest that JPTFY therapy could modulate hyperlipidaemia in HFD-fed obese mice and inhibit cardiovascular complications to some extent. This phenotype indicated that HML had an anti-obesity therapeutic effect. Analysis of the chemical composition of the leaf extracts by multi-chromatographic methods showed the presence of phlorizin, sieboldin, and kumatakenin β-7-O-glucoside. The latter 2 compounds were novel findings for this plant. The presence of a glucose ring structure was indicated this is the basic component of the flavonoids found in these compounds.

Previous studies have confirmed that bioactive compounds extracted from macroalgae, particularly from the green algae *Caulerpa lentillifera*, exhibit significant anti-obesity effects in the Caenorhabditis elegans obesity model. These extracts can markedly reduce fat consumption, triglyceride levels, and lipid droplet marker levels, while simultaneously decreasing the levels of reactive oxygen species in worms fed a high-sugar diet, without affecting their lifespan [42]. As reported, *Malus doumeri* leaf flavonoids exhibit robust anti-oxidative capabilities *in vitro*, capable of mitigating oxidative stress damage induced by H_2_O_2_ in 293 T cells. Consequently, *Malus doumeri* leaf stands out as a natural substance with notable antioxidant properties, hinting at its potential to impact a wide range of diseases [43]. It is also reported that the top 10 of 32 core targets were closely related to obesity-related processes including SRC, HRAS, Rho A, Akt1, and CDC42. Yang *et al.* [44] have reported that the damaged steroid receptor coactivator SRC-1 directly interacted with phosphorylated STAT3 which led to increased food intake and weight gainin a HFD mouse model. The accumulation of c-SRC in the membrane sub-regions can lead to JNK activation, and this participates in physiological processes such as apoptosis and cell proliferation, which is consistent with the predicted results. It has also been shown that JNK was activated during obesity [45]. Oba *et al.* [46] and this indicated the presence of an oncogenic HRAS mutation renders resistance in HFD-induced obesity in fasted mice. Rho A is known to interact with ROCK1/ERK1/2 pathway in order to inhibit adipogenesis [47]. Furthermore, Akt1 and Akt2 play a vital role in maintaining brown adipose tissue [48]. In rat experiments, it was found that cell division cycle 42-interacting protein 4 and Akt activities were downregulated and inhibited in rats fed HFD, respectively [49]. In addition, Wu *et al.* [50] reported that epigallocatechin gallate dramatically enhanced the levels of cyclin A2 and cyclin dependent kinase 2, but inhibited adipogenesis by blocking the mitotic clonal expansion at an early stage of adipocyte differentiation.

We further found that the biological processes were closely related to various phosphorylation events, cell proliferation, apoptosis as well as the activities of various kinases. These were involved in one or more cell components related to the cytoplasmic side of the plasma membrane, cytosol, nucleus and cell cortex. The molecular functions were enriched with processes including ATP binding, non-membrane spanning protein tyrosine kinase activity, receptor binding, nitric-oxide synthase regulator activity, protein serine/threonine kinase activity, receptor signaling protein tyrosine kinase activity, and structural molecule activities.

KEGG pathway analysis of the three compounds of HML was mainly related to pathways and proteoglycans in cancer, viral carcinogenesis as well as PI3K-Akt and FoxO signaling pathways. Recently, Lee and Dong [51] found that FoxO6 promoted lipogenesis and elevated TG-rich very low-density lipoprotein (VLDL-TG) secretion, indicating that FoxO6 may play a significant role in hepatic lipid metabolism. Besides, it was deemed that the PI3Ka-AKT1 pathway was associated with promoting energy expenditure, thus inhibiting weight gain and improving insulin resistance in HFD fed mice. Song *et al.* [52] demonstrated that the phytochemical compound, butein, could promote energy expenditure, inhibit weight gain, and improve insulin resistance in HFD fed mice through the PI3Ka-AKT1-prdm4 axis. *In vivo* and *in vitro* studies found that phytol activated the PI3K/Akt signaling pathway and increased adipocyte numbers in inguinal white adipose tissue depots and improved glucose tolerance in HFD fed mice in an effort to combat obesity [53]. As revealed by Li *et al.*, quercitrin activates the PI3K/Akt signaling pathway by upregulating the expression of Peptidase M20 domain containing 1, thereby improving adipocyte catabolism, correcting reproductive and metabolic abnormalities, and exhibiting therapeutic effects on polycystic ovary syndrome combined with insulin resistance [54]. The 3 bioactive compounds extracted from HML were probably involved with up- or down-regulation of *SRC*, *HRAS*, *Rho A*, *Akt1*, *CDC42* and other genes to either activate or to inhibit PI3Ka-AKT1 and FoxO signaling pathways in order to promote their anti-obesity effects.

Natural anti-obesity compounds mainly include flavonoids (*e.g.*, quercetin, catechins), polyphenols (*e.g.*, catechins, resveratrol), terpenoids (*e.g.*, ginsenosides), organic acids (*e.g.*, malic acid, citric acid), alkaloids (*e.g.*, caffeine, theobromine), and phytosterols (*e.g.*, beta-sitosterol). These compounds regulate fat cell growth, inhibit fat accumulation, improve fat metabolism, and stimulate metabolic processes. In HML, the identified anti-obesity compounds primarily belong to flavonoids (*e.g.*, sieboldin) and glycosides (*e.g.*, kumatakenin β-7-O-glucoside, phlorizin), which regulate key signaling pathways like PI3K-Akt and FoxO, contributing to anti-obesity effects [55-57].

## CONCLUSION

This study is the first to report that hawthorn (*M. doumeri*) leaf extracts have a significant therapeutic effect on obesity. Three bioactive compounds were isolated from these extracts, namely phlorizin, sieboldin, and kumatakenin β-7-Oglucoside. These compounds act through intracellular components such as SRC, HRAS, RHOA, AKT1, CDC42, and CCNA2 in order to interfere with several signaling pathways, including the PI3K-Akt, proteoglycan, and FoxO pathways, so as to achieve their anti-obesity effects.

## STUDY LIMITATIONS

This study is limited by its reliance on a single animal model, which may not fully capture the complexity of obesity in humans. The biochemical analyses focused on serum lipid profiles and did not include comprehensive metabolic or hormonal parameters. In addition, although network pharmacology helped identify potential molecular targets, these interactions were not experimentally validated *in vitro* or *in vivo*. Further research is needed to confirm these mechanisms and evaluate the long-term safety and efficacy of *M. doumeri* leaf extracts.

## AUTHORS’ CONTRIBUTIONS

The authors confirm their contribution to the paper as follows: Writing - Original Draft Preparation: LC. Methodology: GM. Visualization: YP. Investigation: YL. Data Curation: LY. Data Collection: DH. Data Analysis or Interpretation: LJ. Validation: M-SL. Study Concept or Design: WW. Writing - Review and Editing: CL. All authors reviewed the results and approved the final version of the manuscript.

## Figures and Tables

**Fig. (1):**
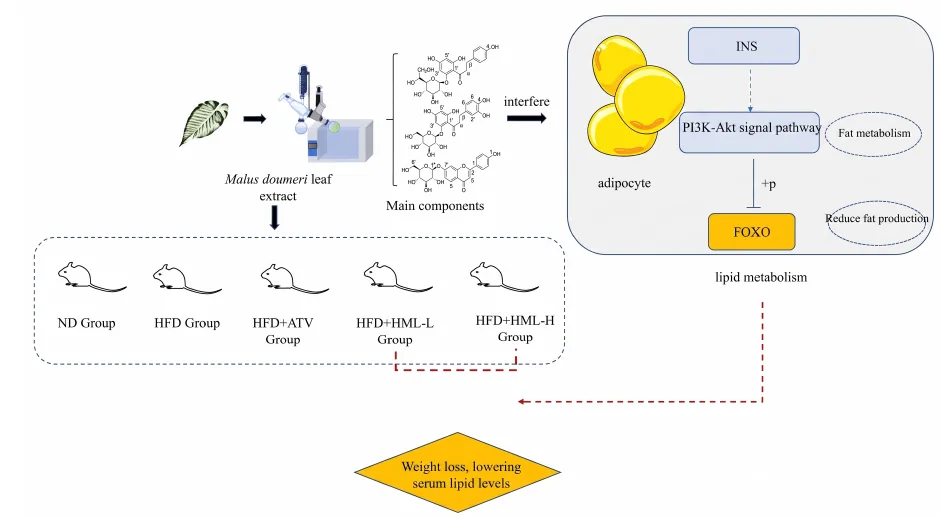
The study process.

**Fig. (2):**
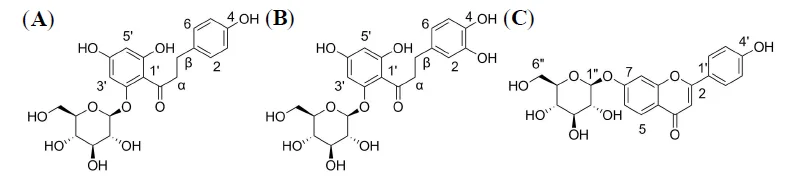
The Chemical structures of (**A**) phlorizin, (**B**) sieboldin, and (**C**) kumatakenin β-7-O-glucoside which were identified from the ethyl acetate extracts obtained from *M. doumeri* leaves.

**Fig. (3):**
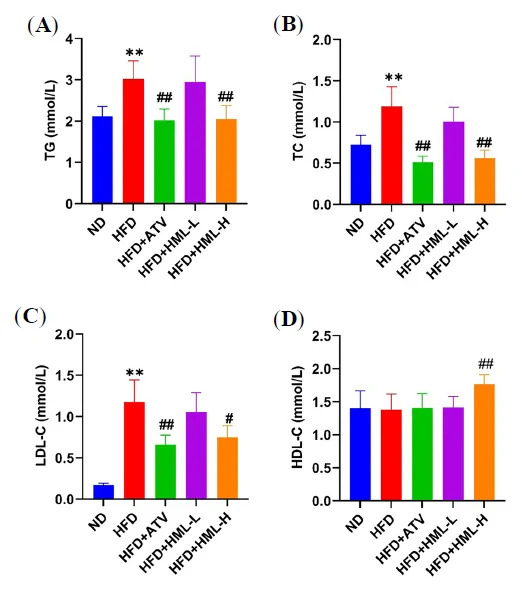
The comparative levels of (**A**) serum triglyceride, (**B**) low-density lipoprotein, (**C**) high-density lipoprotein and (**D**) total cholesterol in the different groups of rats in this study. ** *vs*. ND: P < 0.01; # *vs*. HFD: P < 0.05; ## *vs*. HFD: P < 0.01; ND: normal diet; HFD: high-fat diet; HML: hawthorn (*M. doumeri*) leaves extracts; n = 10 rats in each group.

**Fig. (4):**
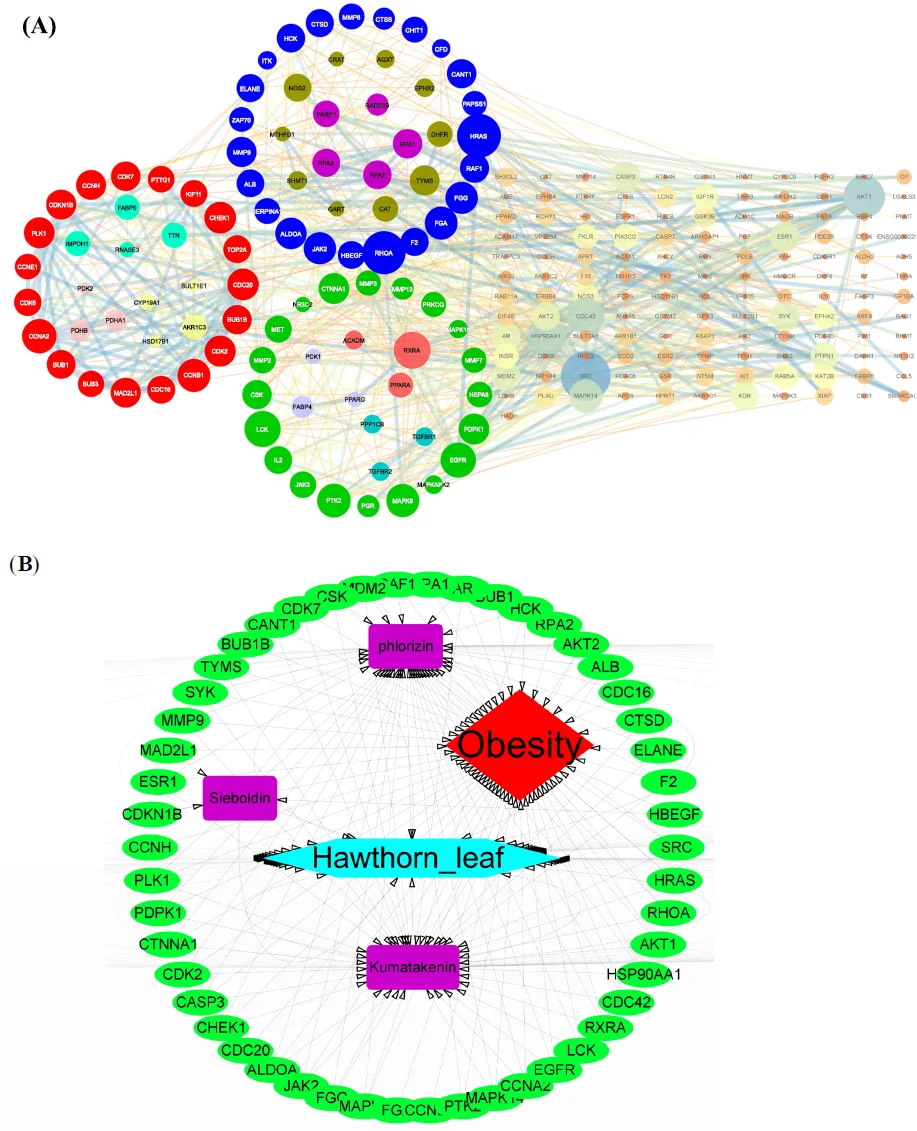
(**A**) The protein-protein interaction and (**B**) chemical-target-disease networks.

**Fig. (5):**
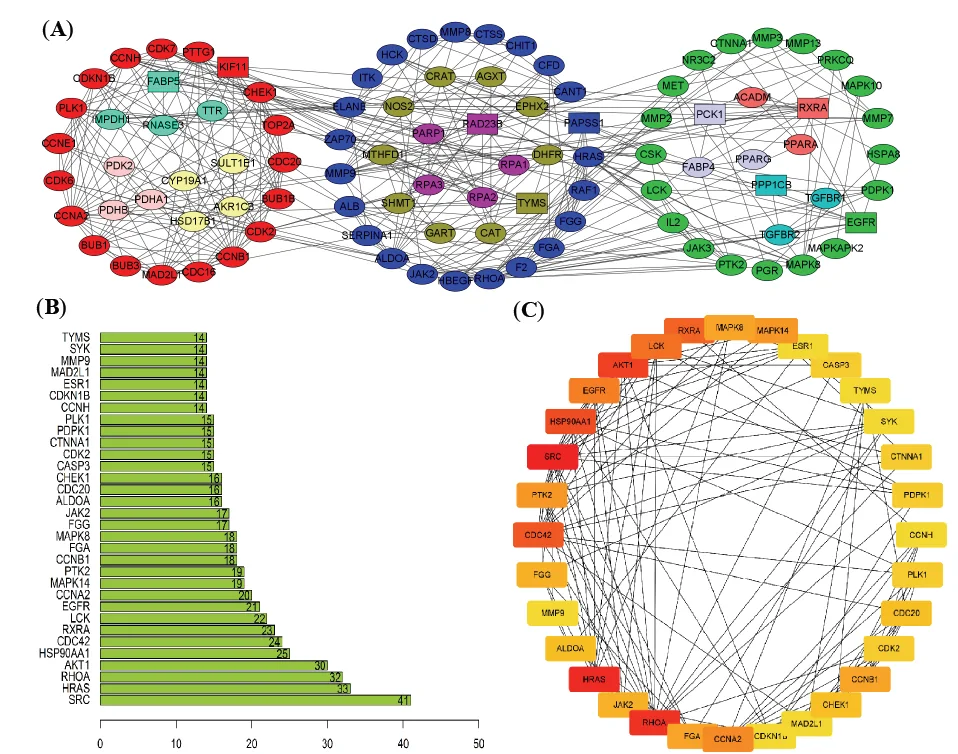
(**A**) Cluster and core target analysis by MCODE and (**B, C**) the cytoHubba algorithms and 32 hub genes of *M. doumeri* leaf extracts with anti-obesity properties.

**Fig. (6):**
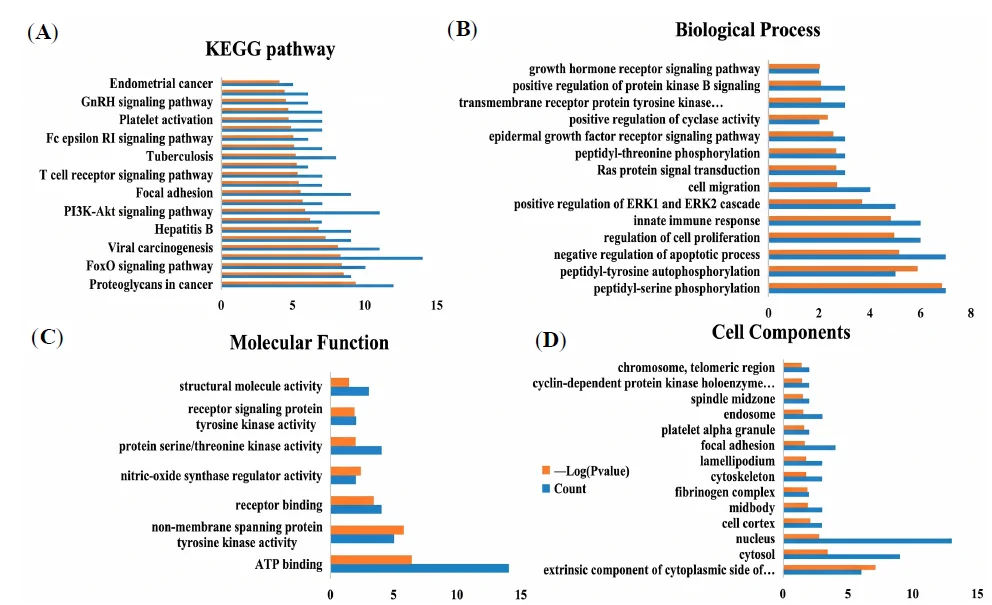
(**A**) KEGG and GO [(**B**) Biological Process, (**C**) Molecular Function, and (**D**) Cell Components] pathway enrichment analyses of 32 core targets.

**Table 1:** Effects of the hawthorn (*M. doumeri*) leaf extracts on the body weight.

**Variables**	**ND**	**HFD**	**HFD+ATV**	**HFD+HML-L**	**HFD+HML-H**
Initial body weight (g)	249.70 ± 3.23	251.70 ± 3.28	249.00 ± 4.40	251.6 ± 3.05	251.6 ± 2.35
Final body weight (g)	352.90 ± 5.71	396.3 ± 3.93^***^	360.10 ± 4.58^###^	360.70 ±3.10^###^	357.20 ± 2.55^###^
Weight gain (g/week)	25.81 ± 1.07	36.15 ± 0.57^***^	27.80 ± 0.19^###^	26.40 ± 1.04^###^	27.26 ± 0.86^###^

**Note:** ***, compared to ND group, *p* < 0.001; ###, compared to HFD group, *p* <0.001.

**Table 2:** The top 10 of core targets with 8 algorithms obtained from cytoHubba.

**Algorithms**	**Closeness**	**BottleNeck**	**Degree**	**DMNC**	**MCC**	**MNC**	**EcCentricity**	**EPC**
NO.1	SRC	SRC	SRC	PTTG1	CCNA2	SRC	ESR1	SRC
NO.2	AKT1	RXRA	HRAS	TOP2A	CCNB1	RHOA	CDK2	RHOA
NO.3	RHOA	AKT1	RHOA	BUB3	CDC20	HRAS	CCNB1	HRAS
NO.4	HRAS	ESR1	AKT1	KIF11	MAD2L1	AKT1	CCNA2	AKT1
NO.5	HSP90AA1	NOS2	HSP90AA1	BUB1	BUB1B	CDC42	HSP90AA1	LCK
NO.6	MAPK8	MAPK8	CDC42	BUB1B	PLK1	LCK	RPA1	HSP90AA1
NO.7	CDC42	HSP90AA1	RXRA	MAD2L1	BUB1	HSP90AA1	CDK6	CDC42
NO.8	RXRA	CYP19A1	LCK	CTSS	TOP2A	CCNA2	CCNE1	PTK2
NO.9	MAPK14	MAPK14	EGFR	RAD23B	PTTG1	EGFR	EPHX2	EGFR
NO.10	LCK	CHEK1	CCNA2	PLK1	KIF11	PTK2	CDKN1B	MAPK8

## Data Availability

The data that support the findings of this study are available from the corresponding author upon reasonable request.

## LIST OF ABBREVIATIONS

BMI: Body Mass Index
BAT: Brown Adipocyte Tissue
FDA: Food and Drug Administration
MMS: Magnetic Mass Spectrometer
NMR: Nuclear Magnetic Resonance
Q-TOF: Microquadrupole Time-of-Flight Mass Spectrometer
ND: Normal Diet
HFD: High Fat Diet
TC: Total Cholesterol
TG: Triglycerides
HDL-C: High-density Lipoprotein Cholesterol
LDL-C: Low-density Lipoprotein Cholesterol
HNL: Human Neutrophil Lipocalin
VLDL-TG: TG-rich Very Lowdensity Lipoprotein

## RESEARCH INVOLVING PLANTS

The mature leaves of *M. doumeri* (Bois) Chev. were purchased from Napo, Guangxi, and identified by Prof. Xindong Nong from Guangxi Botanical Garden of Medicinal Plant. A representative specimen (voucher number: 45102613112-3034LY) has been deposited in the Guangxi herbarium of the Traditional Chinese Medicine Institute for further reference.
